# Supplementary material for: A Retrospective Cross-Sectional Study Assessing Self-Reported Adverse Events following Immunization (AEFI) of the COVID-19 Vaccine in Bangladesh
Source: Vaccines (Basel). 2021 Sep 28;9(10):1090. doi: 10.3390/vaccines9101090 (PMC8538494; doi:10.3390/vaccines9101090)
Supplement: Supplementary file 1 [file vaccines-09-01090-s001.zip › Questionnaire final.pdf]

A Survey on the "After Effect Following Immunization by Coronavirus Vaccine" in Bangladesh

Section 1

The deadly Coronavirus Disease 2019 (COVID-19) has claimed numerous lives and the number is increasing day by day. So, in this situation, a vaccine has been expected to decrease the mortality rate worldwide and save us from this disaster. But there have been some complications reported from the vaccination process, however rare or mild those are. Now that some vaccines have been approved for emergency use, we want to investigate whether these vaccines cause any after-effects. You are cordially invited to participate in this study by providing your valuable response if you have taken at least one dose of COVID vaccine.

* Required

Before you start, let me remind you that the information given by you will be kept confidential. If you have any questions, you may ask us or anyone you feel comfortable with.

For any query or suggestion,

Prof. Dr. Md. Shah Amran
Phone: +8801532603359,
Email: amranms@du.ac.bd

1. I consent voluntarily to participate in this study. *

☐ I Agree

Section 2: Personal Information

2. Your Name *

…………………………………………..

3. Your Age *

………………………………………….

4. Gender (Mark only one oval) *

- Female
- Male

5. Educational Qualifications (Mark only one oval) *

- Primary
- Secondary
- Higher Secondary
- Undergrade
- Graduate
- Post graduate/Doctoral

6. Area of Residence (Mark only one oval) *

- Rural
- Urban
- Foreign

Section 3: Vaccination Information

7. Manufacturer Name: *

……………………………………………………………..

8. Manufacturer Country: *

………………………………………………………………….

9. Vaccination Date: (Example: January 7, 2019) *

…………………………………………………………………….

10. Vaccination Place /Center- *

…………………………………………………………………….

11. Vaccine Dose: (Mark only one oval) *

- First dose
- Second dose

12. Vaccine Dosage Form (Mark only one oval) *

- Injection
- Tablet
- Capsule
- Other: …………………

13. Any specific information was given during vaccination

…………………………………………………………………

Section 4: Health Condition Before Vaccination

14. Were you a confirmed COVID-19 patient before the vaccination? (Mark only one oval) *

- Yes
- No

15. If yes, then what types of medication have you taken? Give in details.

………

16. If yes, were you given the 'Plasma Therapy'?

………

17. If not, did you have COVID-19-like symptoms before the vaccination? (Mark only one oval)

- Yes
- No
- Could not identify

18. Did you have any disease before the vaccination? (If yes, name the disease(s)) *

………………………………………………………………………………………………

19. Were you taking any medications before the vaccination? (If yes, what were those?)

………

20. Have you taken any medication after the vaccination? (If yes, what are those?)

………

21. Do you have any allergy? (If yes, against what?)

………

22. Were you vaccinated before for any other illness? (Mark only one oval)

- Yes
- No

23. Have you taken Pneumonia and / or Influenza vaccine during COVID-19 as a preventive measure to prevent coronavirus infection? (Mark only one oval)

- Yes
- No

24. If yes, did you face any problems then? (Please mention the problems)

………

Section 5: Special Circumstances (For Females Only)

25. Were you pregnant at the time of the vaccination? (Mark only one oval)

- Yes
- No
- Maybe

26. If yes, did you face any abnormalities with the pregnancy after the vaccination? (Mark only one oval)

- Yes
- No
- Maybe

27. Have you been breastfeeding after the vaccination? (Mark only one oval)

- ○ Yes
- ○ No
- ○ Maybe

Section 6: After Effects Following Vaccination

28. Did you get affected by Covid-19 after taking the first dose? (Mark only one oval) *

- ○ Yes
- ○ No
- ○ Maybe

29. During and/or after vaccination, did you face any type of physical discomfort? (Mark only one oval) *

- ○ Yes
- ○ No
- ○ Maybe

Section 7: If you have faced physical discomfort, please answer the following questions.

30. Which of the following you have faced? (Check all that apply) *

- ☐ Pain at the site of Injection
- ☐ Irritation at the site of Injection
- ☐ Swelling
- ☐ Itching
- ☐ Burning sensation
- ☐ Fever
- ☐ Nausea
- ☐ Vertigo (dizziness)
- ☐ Drowsiness
- ☐ Decreased appetite
- ☐ Seizure

- ☐ Intussusception (folding of intestine)
- ☐ Anaphylaxis
- ☐ Body pain
- ☐ Others: ……………………………….

32. When did the problem start? *

………………………………………………………………………………………

33. Do you still have this problem? (Mark only one oval) *

- ○ Yes
- ○ No

34. If yes, how do you manage it?

……………………………………………………………….

35. If no, how did it subside?

……………………………………………………………………………….

36. Have you taken any medicine for the problems? (Mark only one oval) *

- ○ Yes
- ○ No

37. How long did the problem go on?

………………………………………………………………………………………

Thanks for participating
