# Supplementary material for: A Retrospective Cross-Sectional Study Assessing Self-Reported Adverse Events following Immunization (AEFI) of the COVID-19 Vaccine in Bangladesh
Source: Vaccines (Basel). 2021 Sep 28;9(10):1090. doi: 10.3390/vaccines9101090 (PMC8538494; doi:10.3390/vaccines9101090)
Supplement: Supplementary file 1 [file vaccines-09-01090-s001.zip › Supplementary_Table_SI.pdf]

Type of variables	Variables	Outcome	Frequency	Percentage (%)
Socio-demographical variables	Gender	Male	33	66
		Female	17	34
	Age	18-30 years	8	16
		30-60 years	38	76
		>60 years	4	8
	Area of residence	Rural	12	24
		Urban	28	56
		Foreign	10	20
	Educational qualification	Primary	1	2
		Secondary	1	2
		Higher secondary	2	4
		Undergraduate	4	8
		Graduate	17	34
		Postgraduate	25	50
Pre-existing disease condition	Diseases	Allergy	1	2
		Anemia	1	2
		Asthma	3	6
		Ischemia	1	2
		Diabetes	11	22
		Hypertension	4	8
		Hepatic disease	1	2
		Seasonal flu	1	2
		Neurological problem	1	2
	Disease free state	Yes	29	58

		Could not identify	2	4
	Fever	Yes	16	32
	Vertigo	Yes	6	12
	Pain at sight of injection	Yes	24	48
	Drowsiness	Yes	6	12
	Nausea	Yes	3	6
	Burning sensation	Yes	3	6
	Itching	Yes	3	6
	Irritation at the site of injection	Yes	2	4
	Swelling	Yes	2	4
